# The fate of immune complexes in membranous nephropathy

**DOI:** 10.3389/fimmu.2024.1441017

**Published:** 2024-08-08

**Authors:** Jie Xu, Haikun Hu, Yuhe Sun, Zihan Zhao, Danyuan Zhang, Lei Yang, Qingyi Lu

**Affiliations:** ^1^ School of Life Sciences, Beijing University of Chinese Medicine, Beijing, China; ^2^ Qi Huang of Traditional Chinese Medicine, Beijing University of Chinese Medicine, Beijing, China; ^3^ Department of Nephropathy, The Third Affiliated Hospital of Beijing University of Chinese Medicine, Beijing, China

**Keywords:** membranous nephropathy, PLA2R1, THSD7A, immune complexes, autoantibody, complement

## Abstract

The most characteristic feature of membranous nephropathy (MN) is the presence of subepithelial electron dense deposits and the consequential thickening of the glomerular basement membrane. There have been great advances in the understanding of the destiny of immune complexes in MN by the benefit of experimental models represented by Heymann nephritis. Subepithelial immune complexes are formed *in situ* by autoantibodies targeting native autoantigens or exogenous planted antigens such as the phospholipase A2 receptor (PLA2R) and cationic BSA respectively. The nascent immune complexes would not be pathogenic until they develop into immune deposits. Podocytes are the major source of autoantigens in idiopathic membranous nephropathy. They also participate in the modulation and removal of the immune complexes to a large extent. The balance between deposition and clearance is regulated by a wide range of factors such as the composition and physicochemical properties of the immune complexes and the complement system. Complement components such as C3 and C1q have been reported to be precipitated with the deposits whereas a complement regulatory protein CR1 expressed by podocytes is involved in the phagocytosis of immune complexes by podocytes. Podocytes regulate the dynamic change of immune complexes which is disturbed in membranous nephropathy. To elucidate the precise fate of the immune complexes is essential for developing more rational and novel therapies for membranous nephropathy.

## 1 Introduction

Membranous nephropathy (MN), also known as membranous glomerulopathy, is one of the most common causes of nephrotic syndrome that happens among all nationalities and race (1). It is a histopathologically defined glomerular lesion characterized by complement-mediated proteinuria and a diffuse thickening of the glomerular basement membrane (GBM) caused by the immune complexes which are deposited on the outer aspect of the glomerular capillary wall (2). As a type of organ-specific autoimmune disease, MN is relatively specific because it is a non-inflammatory disease. There is few or even no inflammatory infiltration beneath the podocyte where the lesion occurs in MN (3). The pathological changes are mainly caused by the immune complexes consisted of antigens, antibodies and complement components. The histopathologic changes that an MN patient suffers can be featured by light microscopy, electron microscopy, and Immunofluorescence (IF) technic. An MN patient could be diagnosed by the existence of electron dense immune deposits and podocyte foot processes effacement (FPE) under electron microscopy. Light microscopic findings can be stable in early course. As the disease progresses, more rigid and thicker glomerular capillary walls and spike-like projections will appear on PAS-stained and silver-stained sections respectively. IF is usually applied for detection of autoantigen, IgG, and complement component on fixed kidney biopsy tissue sections. The IgG staining can be found around the capillary loop in a fine granular fashion (1). Besides, it helps to classify the MN patients by staining different target autoantigens on biopsy sections clinically (4).

It is the GBM and podocytes that bear the brunt during the process of the disease. GBM and podocytes constitute the glomerular filtration barrier (GFB) together with fenestrated endothelial cells (5). Immune complexes formed under the foot processes of podocytes will perturbate the homeostasis of the GFB. As a result, podocytes get injured and even detached from the capillary wall (6). The GBM becomes thickened and damaged, the filtration system is impaired and eventually, proteinuria happens (1, 7). All these pathological changes could be attributed to the initial formation of the immune complexes, our understanding of which has experienced a long and tortuous odyssey in the past decades of years (8). Not content with just describing the pathological changes clinically, the urgence to explore the underlying mechanism has led researchers to establish appropriate experimental models and that is where Heymann Nephritis (HN) came into play (9). The advantages of HN include, but are not limited to, its reproducibility and its similarities to human MN. Soon it became a popular model to study the disease and a host of results emerged in this period, among which Couser and Hoedemaeker (10, 11) overturned the theory of circulating immune complexes proposed by Edgington and Glassock (12, 13) and proved that immune complexes were formed *in situ*. The change of perspectives from circulating immune complexes to *in situ* immune complexes is a paradigm shift which encourages scientists to search for the native autoantigens in MN. In 2002, Debiec and Ronco (14) first identified neutral endopeptidase (NEP) as the antigen in a rare cohort of patients with alloimmune antenatal MN. Up to date, there have been a dozen of autoantigens being discovered including PLA2R and THSD7A that account for 70% and 3-5% of MN patients, respectively (15, 16). The continued identification of neoantigens is another paradigm shift which indicates that the podocyte is not just the target to be attacked by autoantibodies but also the main source of the autoantigens in MN. In the past, podocytes were only considered to be a part of the GFB. But there is growing evidence that have shown podocytes are not just passively involved in the filtration but the regulation of glomerular homeostasis (17) and a target for therapies (18). In the case of MN, they can regulate the course of the disease by playing a central role in the process of formation, modulation, and removal of immune complexes.

## 2 Immune complexes formation

There are inextricable relationships between the kidneys and the immune system (19). The kidneys can actively orchestrate the regional immune system and conversely, be attacked to injury by the immune system (20, 21). On one hand, under the homeostatic condition, the kidneys play an important role in maintaining homeostasis by production of various hormones and chemokines (22–24). There are also kidney-resident immune cells including dendritic cells (DCs) and macrophages functioning as scavengers and phagocytosis (25–28). On the other hand, the kidneys are a typical target of the immune system when it comes into disordered (29). There are a variety of immune-mediated renal diseases caused by the immune system directly or indirectly (21). The general pathogenesis of direct immune- mediated injuries can be summarized as disorders triggered by immune responses targeting the antigens which exist in the kidneys. Kidney can also be the involved organ affected by circulating immune complexes formed extra-renally. Since the antigens can be present extra-renally or intra-renally, the immune-initiated injuries are different. Thus, the clinical manifestations of various immune renal diseases are distinct (29). With the development of multi-omics technology, properties of the immune complexes in different kidney diseases have been elucidated. Comparative features between MN and other immune-mediated kidney diseases are summarized in **Table 1**. The term MN was first accurately used by Jones (35) in 1957 (referred as membranous glomerulonephritis) to distinguish it from other immune-mediated glomerulonephritis such as minimal change disease (MCD) and focal and segmental glomerulonephritis (FSGS). What makes MN distinct is there is barely inflammatory infiltration as an immune-mediated disease. The reason why patients exhibit symptoms of nephrotic syndrome is because the immune complexes can cause thickening of the GBM and injuries to the podocytes that are the major components of the GFB (36). Therefore, the primary question one may expect would be how the immune complexes form in the first place.

**Table 1:** Comparative features of MN and other immune-mediated kidney diseases.

Disease	Immune deposits position	Serum ICs level	Immunoglobulin type	Antigens	Methodology	Sample	Pathological features	References
LN	Mesangium (class I, II) Subendothelial (class III, IV) Subepithelial spaces (class V)	Moderate to high	IgM, IgA, and IgG	dsDNA, nucleosome, *etc.*	Immunofluorescent spot test	15 of 80 SLE patients	Proteinuria; Microscopic hematuria; Tubular abnormalities; Renal insufficiency, *etc.*	(30)
IgAN	Mesangium	High	IgA, IgG	Galactose-deficient IgA1	MALDI-TOF-MS	278 IgAN patients	Glomerular inflammation; Mesangial proliferation; Tubulo-interstitial fibrosis; Loss of renal function, *etc.*	(31)
MN	Subepithelial	None to low	IgG4, IgG1, IgG3	PLA2R1, THSD7A, HTRA1, *etc.*	WB, MS, IP	26 of 37 patients (PLA2R1), 15 of 154 patients (THSD7A)	Proteinuria, GBM thickening; FPE, *etc.*	(15, 16)
Goodpasture’s syndrome	GBM	None	IgG1, IgG3	α3(IV)NC1, laminin-521	IP, WB, ELISA	57 patients (Collagen), One case report (laminin-521)	Interstitial inflammation; Crescent formation; Pulmonary hemorrhage, *etc.*	(32, 33)
MCD	Podocyte	None to low	IgG	Nephrin, *etc.*	ELISA, IP, WB, IIFT	18 of 62 MCD patients	Proteinuria; FPE, *etc.*	(34)

LN, Lupus Nephritis; MCD, Minimal Change Disease; α3(IV)NC1, α3 chain non-collagen 1 domain of type IV collagen; ELISA, Enzyme Linked Immunosorbent Assay; WB, Western Blotting; IP, Immunoprecipitation; IIFT, Indirect Immunofluorescence Test; MALDI-TOF-MS, Matrix-Assisted Laser Desorption/Ionization Time of Flight Mass Spectrometry.

### 2.1 Components

**Figure 1:**
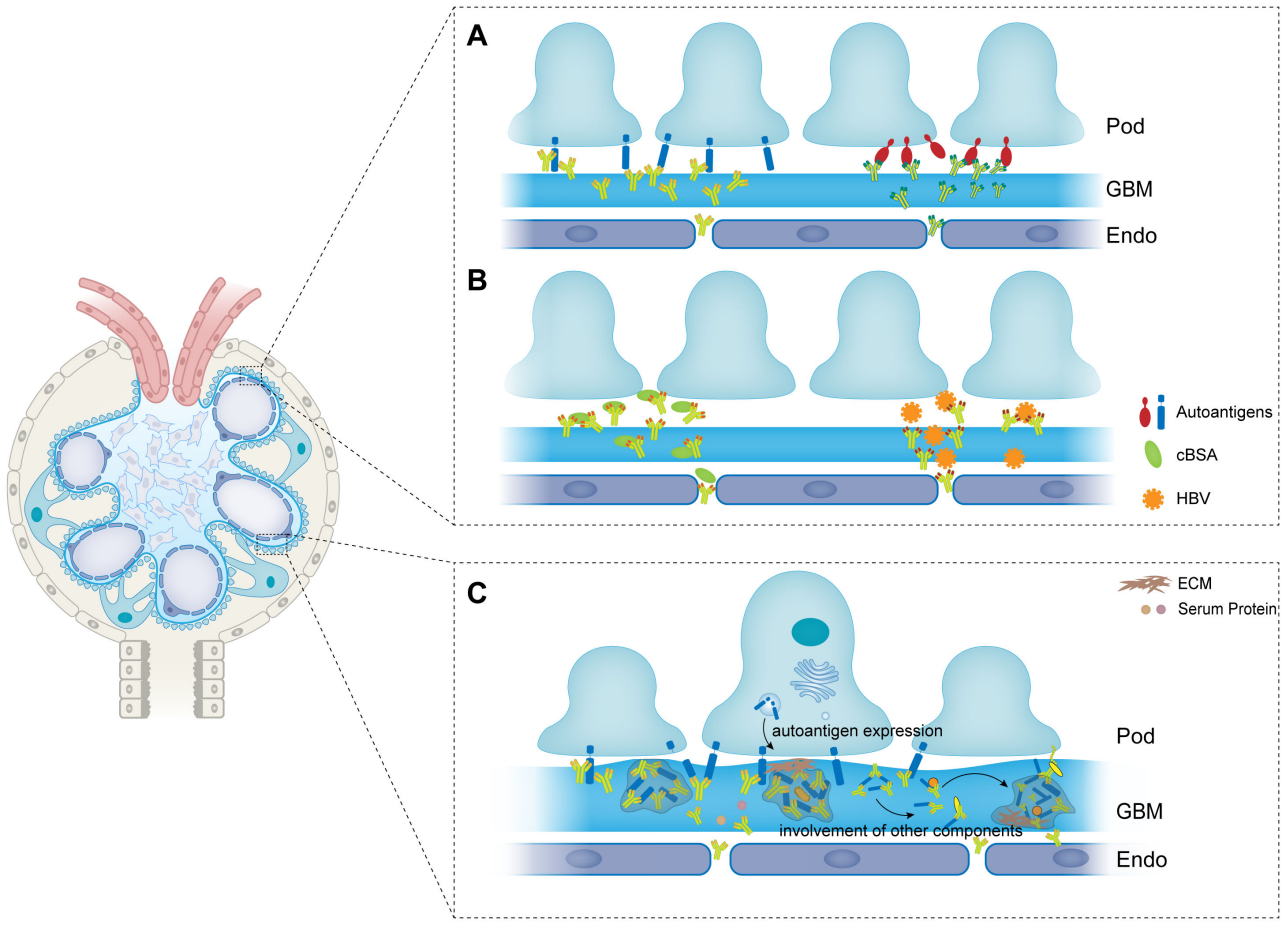
The formation and modulation of immune complexes in membranous nephropathy. **(A)** Autoantigens are expressed on the basal membrane of podocytes, and are recognized by autoantibodies to form immune complexes. **(B)** Exogenous antigens are trapped or planted between podocytes and GBM. Antibodies then recognize and bind to the planted antigens to form immune complexes. **(C)** Antigens and antibodies are aggregated to the formed immune complexes to form larger size of immune complexes. And other components such as ECM, complements and other serum proteins are also involved in the immune complexes. Pod, podocyte; GBM, glomerular basement membrane; Endo, endothelial; ECM, extracellular matrix; cBSA, cationic bovine serum albumin; HBV, hepatitis B virus.

The basic components of the immune complexes are antigens and the corresponding antibodies. There are about 20% of the MN cases are associated with clinical diseases or exposures including systemic lupus erythematosus (SLE) (37), malignancies (38), hepatitis B virus (HBV) (39), and drugs (40). These cases are classified as secondary MN (41). Under this situation, the antigens are usually exogenous molecules that are planted or trapped between the podocytes and the GBM (**Figure 1B**). Cationic bovine serum albumin (cBSA), for example, is a well-studied exogenous antigen in secondary MN. In 1980s, Border and his colleagues (42) established an experimental MN model by immunizing rabbits with cBSA. Based on the fact that the glomerular capillary wall carries negative charges to exert the function as a charge barrier (43), they injected cBSA and control anionic BSA into rabbits intravenously and then perfused the kidneys with sheep anti-BSA antibodies. Rabbits receiving cBSA were found to develop subepithelial immune deposits and severe proteinuria compared to control. Their subsequent work further confirmed cBSA was a type of planted antigen that bound to the anionic sites of the GBM (44). Three decades after the establishment of the model, Debiec and Ronco (45) first reported early-childhood MN in human being. They immunopurified BSA from serum samples of 11 MN patients, 4 of which were children. They found surprisingly that BSA from children could migrate under alkaline conditions while that of adults migrated in neutral pH with no charge. Subepithelial immune deposits containing BSA and BSA specific antibodies (IgG1 an IgG4) were detected on specimen sections of children patients. Cationic BSA is the first antigen reported from food that accounting for the pathogenesis of MN. These discoveries inspire scientists to find other planted antigens in MN. Except for cBSA, there are also HBV (39), hepatitis C virus (HCV) (46), tumor antigens (47), and DNA containing molecules (48) being found in immune complexes in MN. These antigens are either trapped or directly planted on the outer aspect of the GBM and form immune complexes with antibodies and other components.

Corresponding to secondary MN, primary MN (pMN) or idiopathic MN (iMN) refers to those cases that do not have any predisposing factors (49). As shown in **Figure 1A**, Primary MN is a type of autoimmune disease with auto-antibodies targeting autoantigens expressed by podocytes. For many years, the seek for autoantigens in pMN was not going well. After Couser had verified that the immune complexes were initiating by the binding of circulating antibodies to an antigen expressed by the podocytes, in 1982, Farquhar and his colleague (50, 51) identified that the exact pathogenic autoantigen was a glycoprotein with a large molecular weight gp330 (later to morph into megalin). However, megalin is not associated to any human MN cases though it is reported to be expressed in human podocytes recently (52). The first identified antigen in human pMN is neutral endopeptidase (NEP) which is responsible for a rare allo-immune antenatal MN (14). Neonates are born with MN because the mothers developed antibodies during pregnancy which can cross the placenta and target the fetal podocytes. Genetic research demonstrated that the mothers failed to express NEP because there were nonsense mutations or truncating mutations in MME gene coding for NEP (53). Therefore, they develop corresponding antibodies when the fetal NEP proteins appear in the placenta. The antibodies then enter the circulation of the fetus and bind to the NEP proteins that express at the sole of the podocytes (54). The major immunoglobulins produced by the mothers are IgG1 and IgG4 in NEP-related MN of which IgG1 are more pathogenic. Research on clinical cases showed that IgG1 was more necessary for the development of the disease which was detected in the subepithelial deposits whereas IgG4 was not (55, 56).

With the development of laser microdissection and mass spectrometry technology, some neoantigens have been discovered in the past few years (57). The information of the antigens that have been identified so far has been reviewed exhaustively elsewhere (58). PLA2R1 and THSD7A are the first two autoantigens described in adult MN which account for about 70% and 3-5% of the cases respectively. In 2009, Beck and his colleagues (15) reported their seminal work in identifying the target antigens in pMN. They performed Western Blotting of glomerular protein extracts from normal people with serum samples from pMN patients. Antibodies in the serum samples of pMN patients recognized a 185-kDa glycoprotein under non-reducing conditions. This protein was later to be identified as the M-type phospholipase A2 receptor (PLA2R) by mass spectrometry. PLA2R is a type I transmembrane glycoprotein that belongs to the mannose receptor family (59). It has a cysteine-rich domain (CysR), a fibronectin II domain (FnII), and a tandem duplication of eight C-type lectin domains (CTLD 1-8). It was first cloned and identified as the receptor of the secretory phospholipases A2 (sPLA2) in human kidneys in 1995 (60, 61). PLA2R are expressed at human podocytes abundantly and can be found at the basal aspect of the foot processes where the immune complexes are formed. The exact function of podocyte PLA2R in glomerular filtration barrier is not very clear. Some *in vitro* studies demonstrated that PLA2R could enhance the podocyte adhesion to GBM through its FnII domain binding to a major GBM component type IV collagen. Furthermore, the binding is responsible for the collagen-dependent migration (62). The adhesion of podocyte to collagen could be disturbed by serum with anti-PLA2R antibodies (63). The identification of THSD7A took a sincere collaboration of many researchers in the field (16) because the THSD7A-associated iMN cases are relatively rare, only accounting for 5% of those. Similar to PLA2R, THSD7A is a high molecular weight (250 kDa) type I transmembrane glycoprotein. It was initially described by Kuo et al. (64, 65) as a N-glycoprotein that would participate in endothelial migration and tube formation during angiogenesis. THSD7A is also composed of a tandem replication of extracellular domains, there are 21 thrombospondin type I repeat (TSR) domains on its extracellular segment (66). THSD7A is widely expressed on the basal membrane of podocyte like PLA2R. Moreover, its function in the glomerulus is not well characterized either. Subsequent study on THSD7A revealed that it could function as a foot process protein that enhances the attachment of foot process to type IV collagen and stabilizes the slit diaphragm (SD) of podocytes (67). However, whether PLA2R or THSD7A is essential for the intact function of the glomerular filtration barrier remains ambiguous. PLA2R is not expressed on rodent podocytes whereas healthy rodents still have well-functioning glomerulus (68). Also, mouse podocyte specific overexpression of murine PLA2R will not cause any perturbation to the glomerulus (69). As for THSD7A, by knocking down THSD7A in zebrafish, Tomas et al. (70) demonstrated that loss of THSD7A would cause an obvious podocyte phenotype with damaged GFB and protein leakage. On the contrary, it is claimed that THSD7A-deficient mice will not develop any renal phenotype in a recent review (71). Further investigations will be needed to explore the exact role of THSD7A in the glomerular filtration system.

The predominant immunoglobin in PLA2R- and THSD7A-associated iMN cases is IgG4 subclass (72, 73). The interaction between antigens and antibodies will influent the structural integrity and enzymatic activity of the related podocyte proteins and thus bring direct pathogenic effects to podocytes. For example, in PLA2R-related MN, the binding of the corresponding IgG4 to PLA2R will influent its original function—the receptor of phospholipase, whose activation will enhance the release of free arachidonic acid in experimental MN (74). It is also reported that PLA2R can regulate senescence by activating the production of reactive oxygen species (ROS) and stimulating the p53 pathway (75). Thus, the binding of antibodies to PLA2R might potentially contribute the cellular senescence process in podocytes. Since both PLA2R and THSD7A can mediate the adhesion of podocytes to the GBM, the direct targeting of antibodies to PLA2R or THSD7A will interfere with the adhesion and might cause podocyte injury or even detachment from the GBM.

Compared to other human IgG subclasses, IgG4 has some unique properties such as its tendency to undergo Fab-arm exchange process (76) in which anti-inflammatory monovalent antibodies are produced and the resulting limits to form immune complexes with antigens (76, 77). IgG4 is also insufficient to activate the complement system, members of which are verified to be another major component of the subepithelial immune complexes in MN. The activation and regulation of the complement system in renal disease have been reviewed in detail elsewhere (78–81). There are three different pathways that can activate the complement cascade depending on the way of producing C3 convertase, termed the classical pathway, the lectin pathway, and the alternative pathway (82). The classical pathway will be activated when the pattern recognition molecule C1q interacts with the Fc regions of IgM or complement-fixing IgG such as IgG1 and IgG3, which are also detected in nearly all the anti-PLA2R and anti-THSD7A MN cases in addition to IgG4 (83). Haddad et al. (84) reported that the anti-PLA2R IgG4 could directly bind to mannose-binding lectin (MBL), the pattern recognition molecule in the lectin pathway, in a glycosylation-dependent manner. Their work revealed the pathogenic role of IgG4 in anti-PLA2R primary MN and more research is needed to unravel the character of this immunoglobin in all IgG4 dominant MN. All the three pathways involved in complement activating process will lead to the formation of a terminal module, the membrane attack complex (MAC) C5b-9 (85). The insertion of C5b-9 into podocyte membrane will cause cellular injury via a broad of pathways such as the production of ROS (86), protein kinase activation, and stress pathway (87). Consequently, the assembly of the MAC will lead to the proteolysis of a podocyte cytoskeletal protein synaptopodin (SYNPO) and a SD protein NEPH1, and thus, podocyte injury (84). In addition to the cellular injury, the components of the complement system like C1q, C3 and C5b-9 are often detected in the immune complexes in MN. Couser and colleagues have verified that the rat complement C3 is present in the immune deposits along with the sheep anti-Fx1A IgG in pHN (88). They also demonstrated that the rat C5b-9 MAC was detected in the subepithelial immune deposits and the basal side of podocyte membrane close to those in this experimental MN model (89). In the case of human, Arias et al. (90) observed C4d-containing granular GBM deposits in biopsy sections of idiopathic membranous glomerulonephritis and suggested the C4d immunostaining as an auxiliary diagnosis. Chi et al. (91) detected C5a in the subepithelial deposits and introduced it to be a predictor of remission in pMN. A retrospective study of 371 patients in China demonstrated that the intensity of C3 staining was positively correlated with the titers of anti-PLA2R antibody and the stage of pMN (92). In order to identify a more precise spectrum of complement in MN, Sethi group performed mass spectrometry on dissected glomeruli of MN biopsies. Their results showed that a large group of complement related proteins were involved in MN (93).

There are also other components being involved in the immune complexes on the outer side of the GBM. Schneeberger et al. (94) performed a tracer experiment by intravenously injecting tracer proteins including horseradish peroxidase (HRP), catalase and ferritin to pHN rats. Immediately after the injection, the tracer molecules were found to be present in the subepithelial immune deposits. Although the tracers were artificially injected into experimental animals, we could still come to a conclusion that certain substances in the circulation had a chance to be trapped in the immune deposits in MN.

The GBM is a complexed lamellar structure consisting of extracellular matrix components produced by podocytes and endothelial cells including laminins, collagens, nidogens, and heparan sulfate proteoglycans (HSPGs) (95). Podocytes adhere to the outer side of the GBM through interacting of integrin on the basal membrane with laminin (96). By secreting structural and regulatory molecules, podocytes maintain the integrity and homeostasis of the GBM (97). As the bed where the immune deposits implant, the components of GBM would also be present in the immune complexes. Kim and his colleagues (98) studied the differential expression of basement membrane components in MN. They found that the projections of the GBM consisted denser different type IV collagen chains along as another GBM component laminin in stage III MN. Other GBM components were also reported to be presented in the spikes in experimental MN (99). Neither collagen nor the tracers have been identified as antigens in MN despite their existence in the subepithelial deposits. However, their presence is of vital importance for the modulation of immune complexes to immune deposits (**Figure 1C**).

### 2.2 Formation

There have been great advances accompanied by controversies in the understanding of the formation of the immune complexes in MN over the past few decades. In 1959, Heymann and colleagues (9) discovered that the Sprague-Dawley rats developed nephrotic syndrome when they intraperitoneally injected the rats with rat renal suspensions along with complete Freund’s adjuvants (CFA). The disease manifestations were identical to human MN both pathologically and morphologically. Heymann believed that this disease might be due to an iso- or autosensitization reaction although they had not identified the exact responsible antigens. In 1961, Dixon et al. (100) developed the serum sickness nephritis model by daily injecting a slightly excess foreign serum proteins such as human serum albumin (HSA) and bovine gamma globulin (BGG) to albino rabbits. The original model did not conform to the course of human MN because of its rapid response and elimination of the foreign antigens. Their modifications to the model made it a chronic glomerular lesion with immune complexes depositing on the subepithelial aspect of the GBM which is the salient feature of MN. In their modified model, Dixon et al. argued that the serum proteins being administrated to the rabbits had barely affinity to the kidney. Therefore, they claimed that the immune complexes were derived from the circulation and that the glomerulus were just passive victims for their trapping and deposition (101). Since modeling the same clinical disease, researchers tried to apply this theory to the pathogenesis of Heymann’s model. They postulated that antigens in the injection dose of crude kidney homogenate were recognized and bound by corresponding antibodies in the circulation (12, 13). The preformed circulating immune complexes (CICs) were then trapped on the subepithelial side of the GBM when flowing through the glomerulus. It seems reasonable of this speculation considering the kidneys are confronting and filtrating blood with high pressure. However, there are some discrepancies about this theory. It could not explain why the large immune complexes can cross the intact basement membrane and reach its outer aspect. To complement this theory, there was a supplementary theory that the preformed immune complexes would dissociate at the endothelial side of the GBM into independent antigens and antibodies that could permeate through the GBM and re-associated on the subepithelial side (102, 103). The explanation was still flawed because it contradicted the fact that there was barely detection of circulating immune complexes in the serum of neither MN patients nor HN rats.

In 1978, Couser (10) and Van Damme (11) independently developed Heymann’s model into a passive one. They perfused the isolated rat kidneys with heterologous antibodies against the antigens derived from the proximal tubular epithelial cell brush border Fx1A. Results showed that immediately after the perfusion, the anti-Fx1A perfused kidneys developed obvious granular deposits with a diffused pattern compared to the control group. Damme et al. (11) also illustrated that the target antigen was located on the basal membrane of epithelial cells. Using the non-circulating perfusion system, both groups concluded that in HN, the pathological immune complexes were formed *in situ* rather than preformed in the circulation. Subsequent studies consolidate their conclusions. Makker (104) extracted and purified the disease-producing antibody from immunized HN rats and perfused the homologous antibody through isolated kidneys. The antibody was then found to be interacting with antigens expressed on the glomerular capillaries consistent with Couser and Damme’s results. He also showed that the responsible antigen is a mannose containing glycoprotein that was expressed on brush border. Kerjaschki and Farquhar (50, 51) immunoprecipitated the glomerular fractions with anti-Fx1A IgG and purified the precipitate with affinity chromatography. Their results showed that the pathogenic antigen was a membrane glycoprotein with a large molecular weight of 330 kDa (gp330). Their further investigations illustrated that the glycoprotein was an epithelial endocytic receptor called megalin which was expressed on the basal membrane of podocyte foot process facing the outer aspect of GBM (105). The putative ligand binding domains (LBD) of megalin were epitopes-containing domains that were responsible for the recognizing and attacking of circulating antibodies (105, 106). Their novel work had filled the last piece of the puzzle about the pathogenesis of HN which provided profound insights into the investigations of human MN with podocyte proteins acting as autoantigens.

Almost at the same time as Kerjaschki’s publications, Border and his colleagues (42) reported a rabbit model of MN by immunizing rabbits with cBSA. They believed that some foreign molecules could serve as antigens in MN by being planted on the GBM and that electrostatic interaction might be a key factor in this process. Since the GBM is negatively charged, they chose to inject rabbits with cBSA. They found that rabbits developed diffuse capillary wall deposits of IgG and more severe renal lesions compared with those injected with neutral BSA. By perfusing the isolated immunized rabbit kidneys with sheep anti-BSA antibody, Alder et al. (44) confirmed that cBSA was initially planted in the glomerulus followed by *in situ* formation of immune complexes. In fact, as early as in his paper, Dixon (100) had suggested the possibility of exogenous infectious combining with renal tissue and eliciting immune responses. Decades after his statement, the relative animal model was established and decades after the experimental model, the corresponding human MN cases were identified by Debiec and Ronco as we mentioned above. In addition to cBSA, other non-native antigens in MN can also be classified as planted antigens such as DNA complexes in membranous lupus nephritis. The complexes consist of cationic histones which mediates binding of DNA complexes to the GBM by interacting with a GBM constituent heparan sulphate proteoglycan (HSPG) (107).

## 3 Immune complexes modulation

In MN, the subepithelial immune complexes will not persist or turn into immune deposits until they undergo modulation so as not to be cleared by the glomerulus. Nangaku and Couser reminded us that immune complexes were a dynamic interaction process among their components rather than a stationary structure (108). Modification to the immune complexes and dynamic alternation inside would play an important role in the course of MN.

### 3.1 Antigen and antibody properties

The antigens and corresponding antibodies build a lattice-like network together with other components by interacting with each other covalently or noncovalently (109). Physical and chemical properties of these constituents are one of the factors which determine whether the lattice will be cleared or retained as subepithelial immune deposits in MN. These include: size of the immune complexes; subclass, valent, affinity, and charge of the antibody; epitopes and charge of the antigen; and nature of the chemical bonds between antigen and antibody. The size of an immune complex is depending on how many molecules are involved in a single unit. The more molecules an antibody or an antigen can bind, the larger the immune complex will be. The predominant subclass of immunoglobin in MN is IgG, which has a valance of two, meaning that one IgG molecule can bind two monovalent antigen molecules.

Although uncommon, other isotypes of immunoglobin are also involved in the pathogenesis of both iMN and sMN. Lee et al. (110) reported a case of MN with a strong granular staining of IgM in the subepithelial deposits. In this case, the patient was suffering from severe Waldenström’s macroglobulinemia (WM), a lymphoplasmacytic lymphoma with an abnormal expansion of IgM-producing lymphocytes (111). It was believed that occurrence of MN was secondary to WM, paraneoplastic process of which led to the formation of IgM deposits. Zhang et al. (112) reported a secondary MN patient who had a 16-year history of type I diabetes mellitus. A strong staining of IgM containing immune complexes was found with a subepithelial and intramembranous pattern along the capillary wall. Compared to IgG, IgM tends to form pentamers and thus having a capacity for ten antigens which means that IgM containing immune complexes would have a very large size. Not confined to sMN, IgM deposits are also involved in some pMN cases. Hirose et al. (113) reported a 72-year-old case of pMN with monoclonal IgM lambda deposits. Immunostaining on renal biopsy sections showed the patient was THSD7A positive and that a diffuse deposit of THSD7A was along the glomerular capillary walls together with IgM. To investigate the clinical manifestations and outcomes of pMN patients with glomerular IgM deposits, Xu et al. (114) conducted a cohort study enrolled 210 pMN patients with or without IgM deposits in China. Their results showed that patients with IgM deposition were more susceptible to FSGS lesions and presented with more C1q deposits compared with patients without IgM deposition. They also had a significantly worse prognosis with a decreased renal function. Some studies suggested that patients with glomerular IgM deposition presented with more severe renal outcomes because IgM was prone to trigger FSGS and activate the complement system (115–117). However, the molecular mechanism of IgM mediated lesions in MN and whether IgM affects the property and fate of immune complexes in MN remains vague and further studies are needed to work on that.

Like different immunoglobins have different capacity for antigen molecules, the number of antibodies that will bind to different antigens or even the same antigen in different stages of an autoimmune disease will be diverse. The process in which the epitopes of an antigen recognized by the immune system are amplified is known as epitope spreading (118). Epitope spreading is widely involved in autoimmune diseases such as rheumatoid arthritis and systemic lupus erythematosus (119). In MN, epitope spreading was studied both in clinical and in experimental models. Shah et al. (120) investigated the phenomenon in HN by immunizing rats with recombinant megalin N-terminal fragment L6 which was reported to induce severe autoimmunity in HN rats. Sera obtained in the first few weeks only reacted with L6 fragment, whereas with the progression of the disease, the sera could react with all four recombinant fragments spanning other domains of megalin besides L6. Accompanying the presence of epitope spreading was more severe nephrotic syndrome during the disease progression. Epitope spreading in human pMN was illustrated by Seitz-Polski and colleagues (121, 122). It was previously reported that the CysR domain of PLA2R was a major epitope in pMN (123, 124). Polski’s results showed that another two distinct epitopes within CTLD1 and CTLD7 domain were targeted by multi-antibodies as the disease progressed. Patients with more than one epitope were more resistant to therapy and reduced likelihood of remission (121). One possible explanation is that epitope spreading can augment the crosslinking between antibodies and antigens. Crosslinked immune complexes formed by polyvalent auto-antibodies and antigens are more inclined to evolve into stable immune deposits that would not be cleared from the GBM. As a matter of fact, it had been evidenced that polyvalent antigen-antibody interactions were essential for the formation and persistence of the subepithelial electron dense immune deposits compared to monoclonal antibodies in HN (125). The latter were more prone to be cleared from the subepithelial space or even would not immunoprecipitate (126, 127).

### 3.2 Immune complexes rearrangement

According to Nangaku and Couser’s statement, the immune complexes shall undergo a series of rearrangement and dynamic changes of the components before they turn into subepithelial deposits. Sometimes the immune complexes might even undergo dissociation and reassociation during this process. Germuth et al. (128, 129) created immune complexes with diverse affinities by precipitating ovalbumin (OVA) with rabbit antibodies of different avidities *in vitro*. The immune complexes were then injected into mice intravenously. Results showed that mice administrated with high-affinity immune complexes were developing only mesangial deposits while those with low-affinity immune complexes could form subepithelial deposits on the outer aspect of the GBM.

The reassociated immune complexes exhibit more stable physicochemical properties and denser lattice structures which allow them to persist and precipitate along the capillary loop. Experimental support for this mechanism was provided by Mannik and colleagues. By irradiating the immune complexes with light *in vitro*, they produced fixed immune complexes with strong covalent bonds that would not be modified after injected into mice (130). Mannik found that both the irradiated and non-irradiated complexes would interact with the glomeruli in an equivalent manner. But only the non-irradiated immune complexes could persist in the glomeruli and developed into glomerular deposits over time. The cross-linked immune complexes, however, were cleared from the renal system within four days (130).

Another effect of the rearrangement process is, by breaking and re-connecting the interactions among the components, it allows more other ingredients to be involved in the complexes which was raised by Furness as ‘molecular accretion’ theory (131). Incorporation of these constituents turns the original binary antigen-antibody unit into a multicomponent structure which possesses a larger size and more prone to precipitate. As mentioned above, complement factors are another dominant class of components besides antigens and antibodies. Complement components had been reported to interact with circulating immune complexes covalently (132). It was assumed that C3b could break the interactions between Fc domains which was believed to be responsible for the crosslinking and aggregation of the lattice network (133). According to the properties of the immune complexes, the consequence of C3b involvement will be diverse. When the Fc-Fc interactions are interfered, the complexes are dispersed into small pieces that are more soluble. For those immune complexes in circulation, C3b-contained pieces will be captured by erythrocytes and leukocytes through complement receptor (CR) expressed thereon, thus facilitating the clearance of the immune complexes (134). Nevertheless, on the other end of the spectrum, complement components cannot interfere with the interactions between antigens and antibodies. Therefore, when a strong immunoreactivity happens in MN, the small immune complexes are enlarged and reaggregated through interacting with continuously generated antigens and antibodies. During this dynamic process of dissociation and reassociation, the complement components which are supposed to be functioning as scavengers are precipitating together with the immune complexes, making them more sophisticated and multicomponent structures that deposit on the GBM consequently. Deposits with complement will cause more severe damage to the GBM compared to those without complement components (135).

As we have mentioned above, the GBM is not just a passive target of the immune deposits in MN. Components of GBM are also involved in the reassociation of the subepithelial immune complexes. It was reported that the proportion and distribution of GBM components were altered in experimental and clinical MN (98, 99, 136, 137). Collagens and laminins were found to be accumulated between and around the formed immune deposits, disordered distribution of which led to thickening and diffuse projections of the GBM (136, 138). As shown in **Figure 1C**, when podocytes and the GBM are injured by the initial formation of the electron dense deposits, the podocytes release excessive extracellular matrix (ECM) components (139). One could speculate that the subepithelial deposits and the sublytic C5b-9 cause podocytes to release more ECM constituents which cover the immune deposits on the GBM in turn. With the dense ECM network crowding around the deposits, the spikes project from the diffuse GBM (**Figure 1C**). It was evident that some areas of the GBM, especially those with immune deposits on, had an increased permeability which would allow macromolecules in the plasma to penetrate the filter and accumulate at the base of the deposits. Schneeberger’s tracing experiments had verified that molecules in the circulation could cross the GBM and participate within the deposits (94). This process might increase the stability of the subepithelial deposits and perpetuate the disease.

## 4 Immune complexes removal

The GFB used to be thought to act as a size and charge barrier that passively sieves the blood stream flowing through the nephrons (5). However, this perspective has several inadequacies. It cannot explain why molecules that cross the GBM won’t be stuck at the basal of podocytes (140). A growing body of evidence has suggested that the GFB, especially the podocyte, plays an active role in clearing clogged materials in the subepithelial space. In MN, if the undeposited immune complexes or even the immune deposits can be removed appropriately, the insult will be reduced. It has been reported by several studies that immune deposits in MN could be cleared (141). Here we discuss several mechanisms that might be responsible for the removal of immune complexes in MN.

### 4.1 Removal by podocytes

As the outermost part of the GFB, it was assumed that podocytes possessed some unique qualities to clean itself and the filter from the accumulated wastes, thus ensuring an efficacy implementation of the filtrating function (142). With the great advance in imaging technology, manifestations of podocytes in physiological and pathological situations have been revealed by accumulating studies. It was believed that alternations in podocyte motility only occurred under pathological conditions. In nephrotic syndrome, for example, the injured podocytes will exhibit cytoskeleton reorganization and consequent leading-edge retraction or even FPE (143). Recently, there is a growing body of evidence suggesting that the basal membrane of podocytes is motile and dynamic under physiological conditions. Using two-photon microscopy, Brähler et al. (144) observed that podocyte FPs of healthy mice were perturbing in a very slight amplitude along the capillary wall. It was assumed that the purpose of the FPs wiggling was to move the slit diaphragm to remove proteins clogging at the slit (145). In MN, subepithelial immune complexes are sometimes found to be associated with the SD (146). Herwig et al. (67) demonstrated that THSD7A was a SD protein which was targeted by autoantibodies directly between adjacent processes. Immune complexes were consequently formed right at the SD. These observations raise a speculation that in MN, podocytes might get rid of the shedding immune complexes from the slits to maintain the structural integrity of the SD, thus enabling an efficacy operation of the GFB. Evidence supporting this assumption is the presence of immune complexes in the apical membrane domains and the basal space of the FPs in THSD7A-asspciated MN (67, 72). However, there is no direct evidence for this proposal so far.

**Figure 2:**
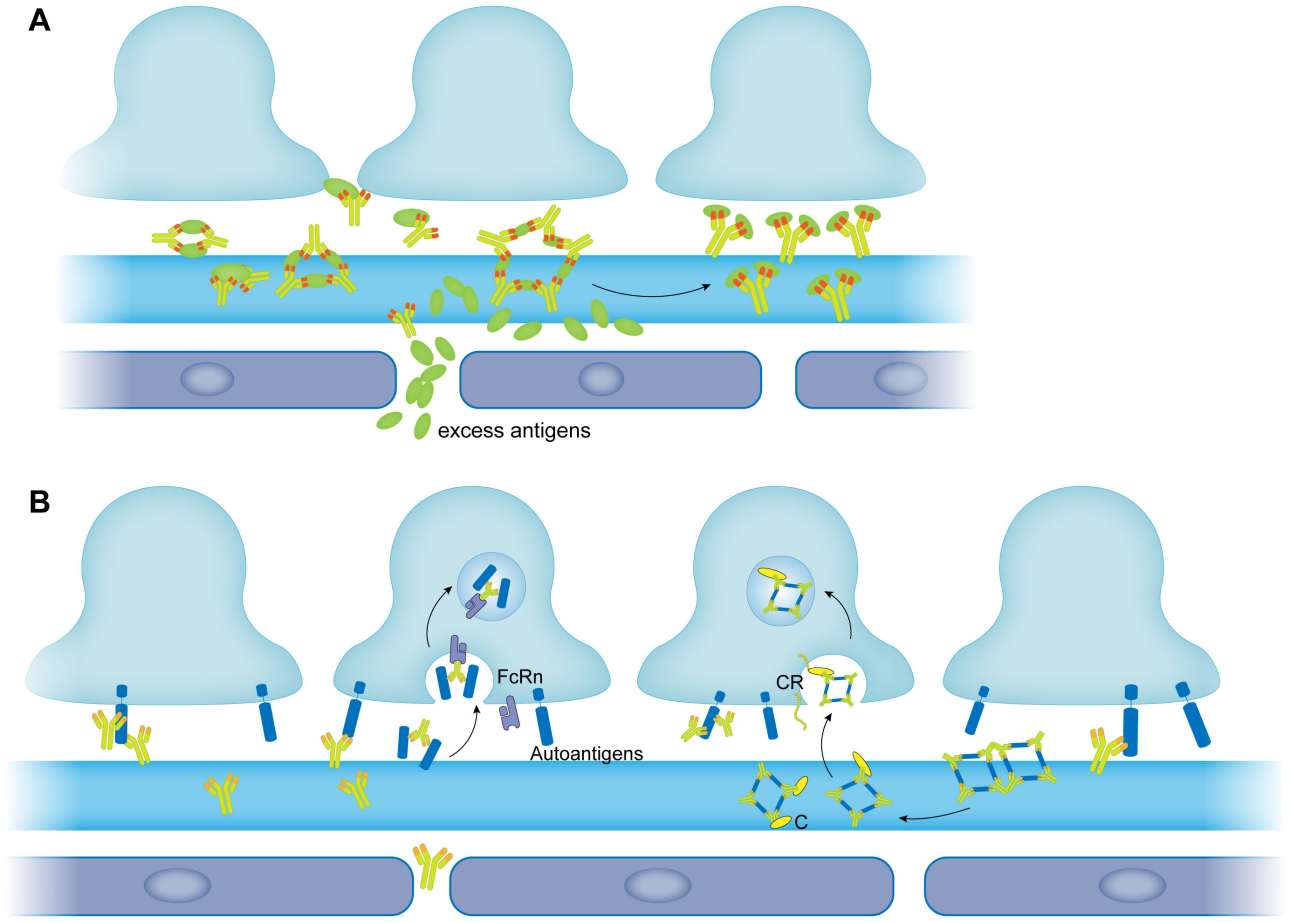
The removal of immune complexes in membranous nephropathy. **(A)** The large size of immune complexes was modulated into small size of immune complexes by excess antigens. **(B)** Immune complexes are removed by podocytes. Immune complexes bind to the FcRn expressed by podocytes and then are endocytosed by podocytes through FcRn (left panel). Immune complexes are modulated by complement into small size of immune complexes. Complement contained immune complexes are then recognized by complement receptor expressed by podocytes and internalized (right panel). FcRn, neonatal Fc receptor; C, complement; CR, complement receptor.

There has been an increasing body of research demonstrating that in addition to being a unit of the barrier, the podocytes also resemble antigen-presenting cells (APCs) in immune mediated glomerulonephritis (147–149). Coers et al. (148) first reported that podocytes expressed MHC class I and II molecules in local inflammation to present antigens and initiate specific T cell responses. Peng et al. (149) illustrated that in IgA nephropathy (IgAN), the IgA deposition would cause podocytes to exhibit macrophage-like properties which is termed podocyte macrophage transdifferentiation (PMT). These observations indicated that podocytes had the potential to remove the immune complexes from the capillary wall like macrophages. Akilesh et al. (150) identified the specific receptor for the clearance as the neonatal Fc receptor (FcRn), which was first described as an Fc receptor responsible for transporting maternal IgG across intestinal epithelium to the fetus (151, 152). Their results showed that podocytes could remove retained IgG from the GBM and transported it to the urinary space by FcRn (**Figure 2B**). Surprisingly, it was reported that podocytes of MN patients expressed more FcRn compared to those of healthy volunteers (153). Haymann et al. (154, 155) verified that podocytes could interact with and internalize aggregated IgG and subepithelial immune complexes both *in vitro* and *in vivo* which indicated podocytes might have a potential to clear immune complexes formed in MN. While on the other hand, Olaru et al. (156) came to an opposite conclusion that FcRn could facilitate the formation of subepithelial immune complexes. They immunized mice with α3NC1, a noncollagenous domain of type IV collagen which had been proved to cause glomerulonephritis in mice. Electron microscopy showed that wild type mice developed extensive subepithelial electron dense deposits and thicken GBM whereas Fcrn^-/-^ mice did not. They assumed the reason was that the FcRn maintained a high level of pathologic antibodies in the serum since it could extend the half-life of IgG. The role of FcRn in pathology is still controversial, especially whether it contributes to the removal of immune complexes in MN requires further investigations.

### 4.2 Removal by complement

Participation of the complement system in the clearance of circulating immune complexes has been well studied (157–159). Basically, immune complexes in the circulation activate the complement system in classical pathway. Complement-opsonized immune complexes then bind to the complement receptor 1 on erythrocytes which transfer the complexes to the liver where the resident macrophages, Kupffer cells, will clear the immune complexes by phagocytosis (160). Besides, the complement system was also reported to be involved in the clearance of tissue-deposited immune complexes especially in the renal system. Furness et al. (161) verified that complement was essential for the removal of glomerular electron-dense deposits in chronic serum sickness glomerulonephritis. Cobra venom factor (CVF) is a complement activating protein which is capable of decreasing complement levels in serum (162). Rats administrated with CVF were found to develop more subepithelial electron dense deposits compared with the control group. Interestingly, removal of the immune complexes did not cease until one week after the complement depletion, indicating that when the antigen-antibody interaction was frequent during the disease progression, opsonization to the antigen-antibody complexes by the complement system was not enough to compensate the reaggregation of themselves. Rather the complement components were precipitated together with the aggregates, which was consistent with our assumption above.

It was reported that podocytes expressed most of the complement components (163) among which CR1 was involved in immune adherence and immune complexes removal elsewhere in the body (131) (**Figure 2B**). Podocyte CR1 was also confirmed to function as an immune adherence receptor (IAR) and to be responsible for the internalization and removal of subepithelial immune complexes in glomerulus (164, 165) (**Figure 2B**). Alexander and colleagues (166) reported a complement regulatory protein in mouse podocytes, complement factor H (CFH) which was analogous to human podocyte CR1. They found that CFH facilitated the removal of subepithelial immune complexes in immune mediated nephritis in mice. Another study conducted by Singh et al. (167) demonstrated that in HN, podocytes would dispose the immune complexes by internalizing them in a complement-dependent manner. Similar mechanism is present in human MN. Mohammad Jalalah detected intracytoplasmic dense inclusions (ICDIs) in podocytes of MN patients in a retrospective ultrastructural study (168). TEM revealed that the ICDIs which exhibited properties of subepithelial electron dense deposits were encircled by the podocyte cytoplasmic processes and retained a minor connection to the GBM. This image captured by TEM illustrated that the inclusions were electron dense deposits that used to distribute along the GBM but endocytosed by the podocytes. The receptor that mediated the internalization was likely to be CR1 since this process did not involve acidic compartments (168) which were coupled with the endocytosis process that FcRn mediated (169).

As we have mentioned above, activation of the complement system in MN usually exacerbates the nephritic syndrome. On the other hand, it was evident that complement components did play a role in the removal of the immune complexes in glomerulus (87). Neutralization of the complement components would ease proteinuria but had little effect on the deposits in MN (88). Studies on CR1 revealed that it participated in both complement regulation and the clearance of the immune complexes and that its expression was decreased in MN (170). Couser et al. (171) administrated a soluble human CR1 on pHN rats and found that sCR1 improved the renal system both functionally and morphologically with decreased proteinuria and immune complexes. Potentially, an appropriate approach which could retain the ability to remove immune complexes of the complement system but avoid their damaging effects would be a promising therapy strategery for human MN in the future.

### 4.3 Removal by excess antigen

*In vitro* studies have demonstrated that excess antigen will lead to the formation of small and soluble immune complexes which tend to be cleared rapidly *in vivo (* 109, 172). The underlying mechanism might be due to saturation of one antibody molecule by different antigens, thus suppressing the formation of lattice network (**Figure 2A**). By administrating an excess dose of antigen to rats, Mannik and Agodoa developed the strategy for removing subepithelial immune complexes in MN induced by cationic HSA (173, 174). Rats injected with excess HSA got a complete removal of immune deposits from their GBM. They also found that cationized HSA was more efficient in the clearance than unaltered HSA, probably because cationized antigens could cross the lamina densa more easily than anionic molecules (174). Once broken into small-latticed units, it would be plausible for the immune complexes to be removed by phagocytosis of podocytes. The authors assumed that if appropriate excess antigen was proposed, it would be feasible for the strategy to be applied in clinical for the dissolution and removal of the immune deposits in MN (174).

## 5 Conclusions

As an immune-mediated glomerular disease, the progression of MN is closely related to the fate of the subepithelial immune complexes. Great advances that illustrate the dynamic changes of the immune complexes in MN have been made in the past decades. The electron dense deposits are formed *in situ* in two distinct processes. In idiopathic MN, circulating autoantibodies target autoantigens which are expressed by podocytes, thus initiating the formation of immune complexes. While in secondary MN, exogenous molecules are implanted in the capillary wall following by targeting of circulating antibodies. A balance between deposition and removal of the immune complexes is established in the glomerulus. On one hand, modulation to the immune complexes occur after their initial formation. With the continuous production of antibodies and incorporation of complement components and other nonspecific molecules, the immune complexes crosslink into lattice networks gradually. If not cleared in time, the lattices will attach to the GBM and turn into electron dense deposits which cause the diffuse thickening of the GBM in MN. While on the other hand, through a variety of mechanisms, the glomerular filtration barrier (GFB) has a novel ability to clear the subepithelial immune complexes. The podocytes, for example, are involved in the removal due to their motility and receptors they expressed. Some factors are double-edged for the fate of immune complexes in MN. For example, complement components are often involved in the immune complexes, whereas complement deficiency also impairs the opsonization, modulation and removal of the immune complexes in MN. The same applies to antigens in MN. Excess antigens will break the immune complexes lattice into small pieces which are more prone to be removed. However, antigens are the main components of the immune complexes which are pathological. The fact that administration of excess antigens can remove immune deposits is only limited to animal experiments. To summarize, factors that exacerbate the crosslinking of immune complexes are pathological, whereas factors which are capable of uncouple the crosslinking of immune complexes are beneficial for the disease.

In this review, we retrospect the two paradigm shifts during the course of research on the fate of immune complexes in MN. We also enumerate the components of immune complexes including antigens, antibodies and complement factors. The process of modulation is critical for the fate of immune complexes which determine whether the immune complexes would be cleared or turn into deposits. In membranous nephropathy, the removal capacity of the glomerulus is overloaded. Immune complexes that are not removed in time will turn into immune deposits and cause damage to the GFB. Lesions of the GBM and podocytes will eventually induce nephrotic syndrome. To prevent the formation of the subepithelial immune deposits is the central idea in the treatment of MN (141). Thus, it will provide us some novel insights into the therapeutic invention of MN to better understand the fate of the immune complexes. The future research should focus on the strategies to modify and remove immune complexes in MN. Hopefully it will provide precision treatment for those refractory MN patients.

## Author contributions

JX: Writing – original draft, Writing – review & editing. HH: Conceptualization, Writing – original draft, Writing – review & editing. YS: Investigation, Writing – review & editing. ZZ: Investigation, Writing – review & editing. DZ: Data curation, Supervision, Writing – review & editing. LY: Funding acquisition, Supervision, Writing – review & editing. QL: Funding acquisition, Supervision, Writing – review & editing.
